# Fundamental difference in life history traits of two species of *Cataglyphis *ants

**DOI:** 10.1186/1742-9994-3-21

**Published:** 2006-12-15

**Authors:** Markus Knaden, Rüdiger Wehner

**Affiliations:** 1 Institute of Zoology, University of Zürich, Winterthurerstrasse 190, CH-8057 Zürich, Switzerland; 2 Department of Evolutionary Neuroethology, Max-Planck Institute for Chemical Ecology, Hans-Knoell Strasse 8, D-07745 Jena, Germany

## Abstract

**Background:**

The two sympatric species of Tunisian desert ants, *Cataglyphis bicolor *and *C. mauritanica*, do not exhibit any differences in their foraging ecology, e.g. in food preferences and in their spatial and temporal activity patterns. Here we show that instead the two species markedly differ in their life histories.

**Results:**

We analysed mtDNA of specimens that were collected along a 250-km transect. *C. bicolor *exhibited a genetically unstructured population (with the genetic and geographic distances among colonies not being correlated). On the contrary the populations of the polygynous *C. mauritanica *were clearly structured, i.e. exhibited a strong correlation between genetic and geographic distances. This difference is in accordance with large queen dispersal distances due to far-reaching mating flights in *C. bicolor *and small queen dispersal distances due to colony foundation by budding in *C. mauritanica*. Furthermore, wherever we found populations of both species to coexist within the same habitat, the habitat was used agriculturally. Mapping nest positions over periods of several years showed that plowing dramatically decreased the nest densities of either species.

**Conclusion:**

We conclude that owing to its greater queen dispersal potential *C. bicolor *might be more successful in quickly re-colonizing disturbed areas, while the slowly dispersing *C. mauritanica *could later out-compete *C. bicolor *by adopting its effective nest-budding strategy. According to this scenario the observed sympatry of the two species might be an intermediate stage in which faster colonization by one species and more powerful exploitation of space by the other species have somehow balanced each other out. In conclusion, *C. bicolor *and *C. mauritanica *represent an example where environmental disturbances in combination with different life histories might beget sympatry in congeneric species with overlapping niches.

## Background

In the highland steppes of Tunisia the two large desert ants, *Cataglyphis bicolor *and *Cataglyphis mauritanica*, occur sympatrically. A recent ecological comparison revealed that the two coexisting species do not show any differences in the use of the three resource characteristics habitat, time and food [1], i.e. in the main factors for which organisms compete [2]. In order to explain the starting point of the study described here, let us briefly conclude the main results of the ecological comparison. Both species have equally sized monomorphic workers (e.g. head width, *C. bicolor *1.7 mm, S.D.: 0.4 mm (n = 500), *C. mauritanica*: 1.6 mm, S.D.: 0.5 mm (n = 500)). Being thermophilic scavengers searching for arthropod corpses, *C. bicolor *and *C. mauritanica *rely on the same food sources without any differences either in the type, size, or dry weight of the collected food items. The workers of both species employ the same individual foraging strategies, i.e. depart from the nest for the same distances, do not occupy food territories, and do not avoid the vicinity of conspecific or allospecific *Cataglyphis *nests. We never observed any kind of conspecific or allospecific interference competition for food. *C. bicolor *and *C. mauritanica *inhabit the same microhabitat, as far as physical ground structure and vegetation is concerned, and they exhibit the same daily activity patterns (for a full description of the ecological analyses see [1]).

The 'Competitive Exclusion Principle' [3,4] based on the classical mathematical model of Volterra [5] states that *n *species cannot coexist on less than *n *resources. Even though this principle has been shown to be violated by numerous studies, it has also been shown that stable coexistences usually require either mechanisms that increase the number of resources present (e.g. feeding on different parts of one plant species, [2,6]), or different impacts on the resource population by coexisting populations [7], or spatially structured habitats [8,9], or interference competition for food [10,11], or different feeding strategies [12], or different life histories of the coexisting populations [13], or instable habitats [14,15], or any combination of these preconditions. Most of these factors do not seem to be responsible for the coexistence of *C. bicolor *and *C. mauritanica *[1]. However, the temporal stability of the habitat and the life histories of the two species in question might well be.

Contrary to the 'Competitive Exclusion Principle' the 'Unified Neutral Theory' [16] explains complexity of ecological communities with ecological equivalence. Hence, following this model niche differentiation is not a prerequisite of the coexistence of species. The "niche" perspective and the "neutral" perspective have now been discussed not to negate each other, but to present the endpoints of a continuum [17]. The aim of the present account is not to test for one of these alternative theories, but rather to identify factors that might explain the coexistence of *C. bicolor *and *C. mauritanica *following the "niche" perspective. If no such factors are found, the "neutral" perspective – of course – has to be revisited.

Even though both species do not differ in their foraging ecology, they do so in their social structure: Whereas *C. bicolor *is monogynous, *C. mauritanica *is truly polygynous [18], a difference that may directly influence the genetic population structure of the two species. While monogynous queens usually found their nests independently after having performed far-reaching mating flights, most of the polygynous species disperse by budding with queens establishing new nests close to their mother colony [19,20]. This distinction leads to strong differences in single-generation migration distances and, as a consequence, to genetically unstructured populations in monogynous species and structured populations in polygynous species [20-22]. However, there are exceptions: The monogynous *Cataglyphis cursor*, which produces new queens via parthenogenesis [23] exhibits the budding type of nest foundation [24].

Different dispersal strategies of the queens in *C. bicolor *and *C. mauritanica *could lead to a better understanding of the coexistence of the two species if, in addition, the temporal dynamics of the ants' habitat are considered. The places, at which *C. bicolor *and *C. mauritanica *live sympatrically, are areas used for farming. Due to the nutritional low productivity of the North African highland steppe regions, wheat is produced only every 5–7 years, while during the rest of the time the land is used for sheep farming. As we never were able to find *Cataglyphis *colonies on freshly plowed areas, plowing most likely destroys pre-existing *Cataglyphis *colonies.

We now hypothesize that the coexistence of *C. bicolor *and *C. mauritanica *is due to the ephemerality of the habitat shared by the two species. Under the assumption that *C. bicolor *is a typically fast dispersing monogynous ant, it could act as a pioneer species on freshly ploughed and hence *Cataglyphis*-free areas. If, on the other hand, *C. mauritanica *is a typically slow dispersing polygynous species, it would need a longer time to reach the ploughed fields. However, once *C. mauritanica *queens accompanied by a group of workers have reached such fields, they could out-compete single *C. bicolor *queens at the available nesting sites. It is well known that dependent nest-founding strategies have higher competition efficiencies than independent ones. When digging the nest, queens of polygynous species are assisted by nestmates and, therefore, face a smaller risk of getting killed by predators or ants of established colonies nearby [20]. Furthermore, polygynous colonies are able to increase colony longevity by queen replenishment [25,26]. In this case, the local sympatry of *C. bicolor *and *C. mauritanica *could be seen as a temporary rather than permanent condition, which would result in pure *C. mauritanica *populations, whenever human agricultural interference should cease to occur.

To substantiate this claim, we shall focus on the question of whether *C. bicolor *and *C. mauritanica *disperse as predicted for monogynous and polygynous species of ants and hence exhibit unstructured and structured population characteristics, respectively. In order to obtain phylogeographic information, we collected ants of both species along large-scale transects and created intraspecific haplotype networks using mitochondrial sequences of the Cytochrome Oxidase I and II genes. In addition, by geographically mapping the haplotypes of both species on 6 small-scale focus areas (about 1.6 – 12.3 × 10^5 ^m^2 ^each) within a 3-year period, we inquire about whether the habitat and thereby the coexisting populations of either species are rather stable.

## Results

In order to test whether the *C. mauritanica *population is genetically structured, we sequenced mtDNA of ants that had been collected along a large-scale transect covering a total length of 250 km.

### Genetic population structure of *C. mauritanica*

**Figure 1:**
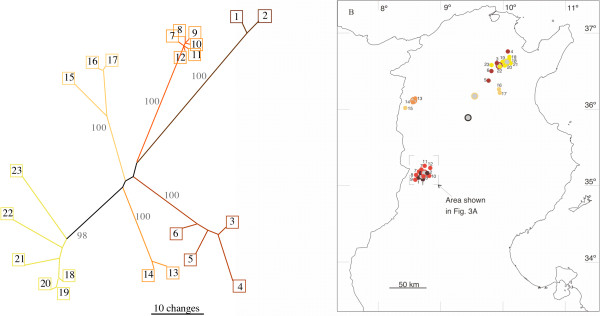
**Phylogeographic distribution of *C. mauritanica *haplotypes in Tunisia**. a. Haplotype network of *C. mauritanica *inferred from mtDNA data. 1044 bp of 1301 bp were constant. The numbers of the bootstrap replicates are 10000. b. Geographic distribution of the different haplotypes. Each small circle represents a colony collected in 2001 for which the haplotype (indicated by the accompanying small numbers) was recorded. Large coloured circles depict the geographic centers of the clades (GCC). The black circle indicates the geographic center of the whole population (GCP).

Along the 250-km transect we found 23 different haplotypes in 28 *C. mauritanica *nests (see Table 1). This large number comes along with a remarkable variability between these haplotypes. 185 of 1301 bp were variable. We found a maximum of more than 5% substitutions between pairs of haplotypes. According to Templeton et al. [27] we constructed a 1-step cladogram (not shown). However the resulting haplotype network revealed 162 haplotype states that would have been necessary intermediates, but non of them was present in the sample of 23 haplotypes mentioned above. Therefore, a nested clade analysis [27,28] failed. However, if the geographic distribution and the haplotype relatedness are compared [29], a clear-cut result emerges: haplotypes belonging to the same clades in the haplotype network occur at small geographic distances from each other (Fig. 1). The distances of the individual samples from the Geographic Center of the Clade (GCC) and from Geographic Center of the Population (GCP) were 11.4 (+/- 18.6) km and 94.9 (+/- 16.6) km respectively (n = 28, Wilcoxon-matched-pairs test, p < 0.0001). In accord with this result the geographic distance and the genetic distance, i.e. the number of substitutions, are correlated (p < 0,05, Mantel-Test [30]). Hence we conclude that the single-generation migration distance of *C. mauritanica *must be rather short.

**Table 1:** List of material examined in present study.

	Haplotype	GenBank nos.	
		CytOxidase part 1	CytOxidase part 2

*C. bicolor*	1	AY642288	
	2	EF139822	
	3	AY642290	
	4	EF139823	
	5	EF139824	
	6	EF139825	
	7	AY642294	
	8	EF139826	
	9	EF139827	
	10	EF139828	
	11	EF139829	
	12	EF139830	
	13	EF139831	
	14	EF139832	
	15	EF139833	
	16	EF139834	
	17	EF139835	

*C. mauritanica*	1	EF139775	EF139798
	2	EF139776	EF139799
	3	EF139777	EF139800
	4	EF139778	EF139801
	5	EF139779	EF139802
	6	EF139780	EF139803
	7	EF139781	EF139804
	8	EF139782	EF139805
	9	EF139783	EF139806
	10	EF139784	EF139807
	11	EF139785	EF139808
	12	EF139786	EF139809
	13	EF139787	EF139810
	14	EF139788	EF139811
	15	EF139789	EF139812
	16	EF139790	EF139813
	17	EF139791	EF139814
	18	EF139792	EF139815
	19	EF139793	EF139816
	20	EF139794	EF139817
	21	EF139795	EF139818
	22	EF139796	EF139819
	23	EF139797	EF139820

In order to test whether this short migration-distance is due to nest budding, we determined the mitochondrial haplotypes of 79 individuals of 79 *C. mauritanica *nests at 5 focus areas, and found a total of 5 different haplotypes (see Table 2). When single areas had a sufficient number of *C. mauritanica *nests and more than one haplotype, as it was the case in areas a, c, and d, the haplotypes showed a clumped distribution (p < 0.05, Mantel-Test [30]). In conclusion, the genetically structured populations as inferred from both the large-scale transect and the small-scale analyses, clearly point at slow dispersal mechanisms, most probably by budding in *C. mauritanica*. Of course, mt-DNA analyses inform only about the dispersal of the queens, but as in ants the males do not contribute to nest founding, in our present case information about male dispersal distances is insignificant.

**Table 2:** Numbers of *C. mauritanica *haplotypes occurring within 5 focus areas near Kasserine (Tunisia) in 2001 (2004).

*C. mauritanica *haplotype							
Area		1	2	3	4	5	unidentified
	
	a		13 (13)		6 (6)		3 (2)
	b					14	4 (9)
	c			3 (1)	12 (12)	1	1
	d	5				21 (8)	31 (5)
	e	2 (6)			2		1 (2)

For the locations of areas a-e and of the haplotypes within these areas see Figs. 3A and 3B, respectively.

### Genetic population structure of *C. bicolor*

**Figure 2:**
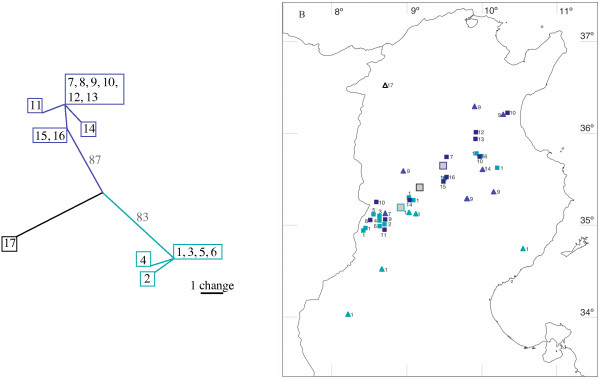
**Phylogeographic distribution of *C. bicolor *haplotypes in Tunisia**. a. Haplotype network of *C. bicolor *inferred from mtDNA data. Haplotypes that revealed no changes of basepairs in the network (e.g. haplotypes 1, 3, 5, and 6) differed in the non-coding region, but due to the impossibility to align this region it was not used for the calculation of the haplotype network. 711 bp of 771 bp were constant. b. Geographic distribution of the different haplotypes. Each small square represents a colony collected in 2001 for which the haplotype (indicated by the accompanying small numbers) was recorded. The triangles depict samples that were collected in 1999 but were included into the phylogeographic analysis. Large coloured squares depict the geographic centers of the clades (GCC). The black square indicates the geographic center of the whole population (GCP).

We sequenced mtDNA of *C. bicolor *ants, collected along the same 250-km transect and within 4 of the 6 small-scale focus areas mentioned above for *C. mauritanica *(2 of the 6 areas mentioned above contained pure *C. mauritanica *populations). Along the large-scale transect we found 16 different haplotypes within a sample of 25 *C. bicolor *colonies. Twenty of the 1217 bp were variable with a maximum of 1.5% substitutions between pairs of haplotypes (see Table 1). Again a nested clade analysis failed because of the lack of 17 intermediate haplotypes between the two main clades of the network. We extended the sample size of the *C. bicolor *transect to 38 nests by adding samples we had collected two years before, but of which we had only CO1 sequences. However, the phylogenetic analyses of either the 771 bp of the CO1 genes or the 1217 bp of the CO1 and the CO2 genes did not lead to any contradicting result. As in *C. mauritanica *we tested for a genetic structuring of the population by calculating the distances of every individual from the geographic center of its clade (GCC) and from the geographic center of the whole population included in the analysis (GCP). In contrast to *C. mauritanica*, in *C. bicolor *there was no difference between the two geographic distances (Fig. 2a, mean distance from GCC: 40.2 +/- 32.2 km, mean distance from GCP = 44.4 +/- 31.9 km, n = 38, Wilcoxon-matched-pairs test, p = 0.18). Correspondingly, there was no correlation between geographic distance and genetic distance of the samples (p = 0.57, Mantel-Test [30]). The appearance of identical haplotypes in distances of more than 180 km (Fig. 2b: haplotypes 1 and 10) is striking, but due to the high frequency of at least one of the two haplotypes in the whole population, these long distances cannot be taken as a proof of long single-generation migration distances. However, the results obtained in the 5 small-scale focus areas lend further support to the hypothesis of long migration distances in *C. bicolor*. We found a total of 7 haplotypes (see Table 3). Again, individuals of most of the nests carried haplotype 1, and hence by themselves do not reveal any further information. Nevertheless, whenever we found nests belonging to the other 6 haplotypes, the latter did not form large clusters, but appeared either individually or close to maximally two or three adjacent nests. These small clusters are most likely caused by the polydomy of *C. bicolor*, i.e. by the fact that colonies usually consist of a queenright mother nest and a few neighbouring queenless satellite nests [31]. The absence of large clusters of nests belonging to the same haplotype speaks against any small-scale dispersal strategy employed by *C. bicolor*.

**Table 3:** Numbers of *C. bicolor *haplotypes occurring within 4 focus areas in 2001 (2004).

*C. bicolor *haplotype									
Area		1	2	3	4	7	8	9	unidentified
	
	c	2					4		(1)
	d	12 (1)				1	1 (2)		9 (1)
	e	19 (10)	1 (2)	1 (1)	1 (1)			3 (1)	2 (9)
	f	17			1				(17)

**Figure 3:**
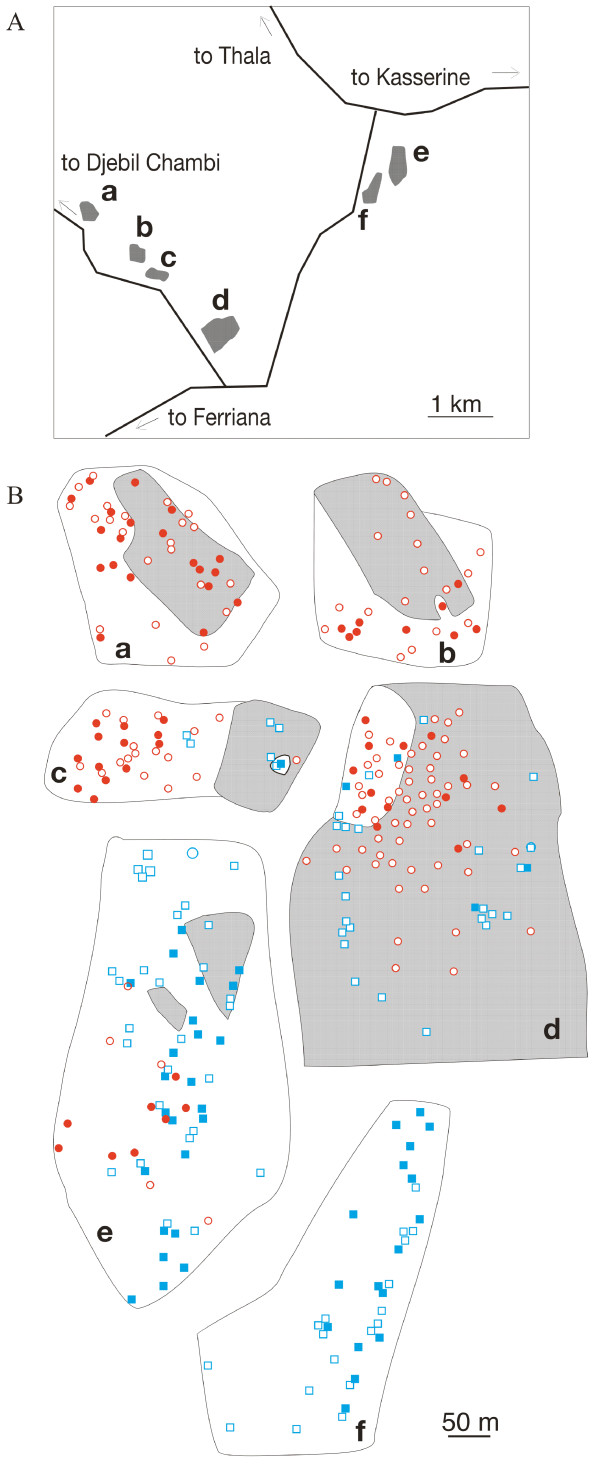
**Effect of human agricultural interferences on the nest distributions in *C. mauritanica *and *C. bicolor***. a. Map of the locations of the 6 small-scale focus areas (dark grey, a-f). For geographic position of the entire area see Fig. 1b. b. Nest distributions of *C. mauritanica *(red circles) and *C. bicolor *(blue squares) in the years 2001 (open symbols) and 2004 (filled symbols). Areas that had been used agriculturally during the 3-year test period are shown in light grey.

For the locations of areas c-f and of the haplotypes within these areas see Figs. 3A and 3B, respectively.

### Habitat longevity

Having shown that *C. bicolor *and *C. mauritanica *differ markedly in the dispersal strategies of their queens, we next focused on the question, whether the local sympatry of the ecologically equivalent *Cataglyphis *species might be a transitional phenomenon due to the temporal instability of the habitat. Therefore we revisited the 6 small-scale areas three years after our first survey (Fig. 3). We again recorded the positions of all *C. bicolor *and *C. mauritanica *nests. In total, the number of nests within these areas had decreased from 193 to 109 (in *C. mauritanica *from 119 to 63, in *C. bicolor *from 74 to 46, see Table 2 and 3, respectively). We also mapped the acreages that had been used for growing wheat during the time period of 2001–2004 (15.2 ha of the total of 37.8 ha of the 6 focus areas revisited in 2004, Fig. 3B). At places that had been farmed, at least during the preceding year, the number of *C. mauritanica *nests had decreased dramatically from 64 to 16, while on the unused areas the decrease was less pronounced (from 55 to 47 nests; χ^2 ^= 14.79, df = 1, p < 0.001). Agricultural activity similarly affected *C. bicolor*, for which the number of nests was only slightly reduced, from 47 to 42 on the unused area, while there was again a dramatic reduction in the number of nests from 27 to 4 on the agriculturally used areas (χ^2 ^= 12.71, df = 1, p < 0.001).

**Figure 4:**
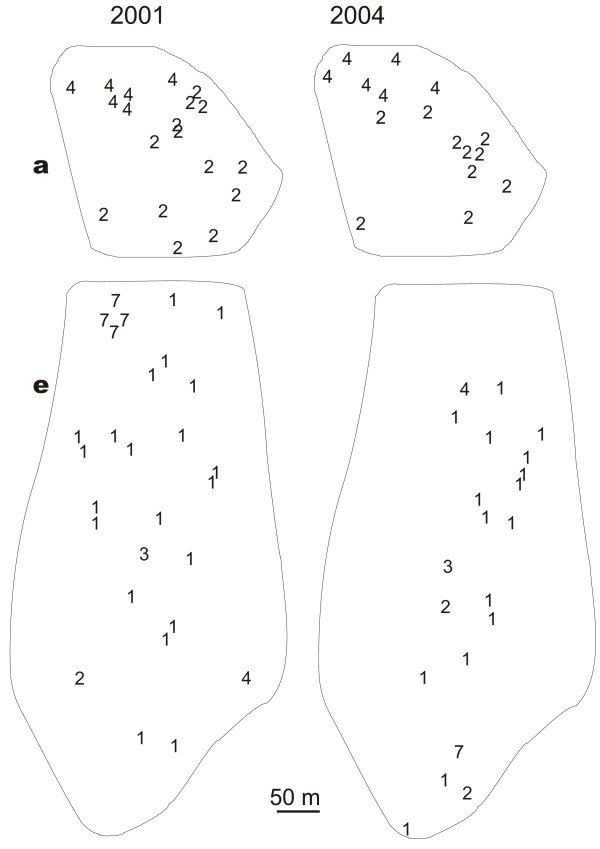
**Distributions of haplotypes within two focus areas (a and e, see Figure 3b) in 2001 and 2004**. a. *C. mauritanica *haplotypes in area **a **in 2001 and 2004 e. *C. bicolor *haplotypes in area **e **in 2001 and 2004. Only nests with identified haplotypes are shown.

The distribution of the mtDNA haplotypes did not vary significantly between the two years 2001 and 2004. All *C. mauritanica *haplotypes present in 2004 had also been found already in 2001. Furthermore, the distribution of haplotypes within the focus areas had remained almost constant (Fig. 4a). The same is true for *C. bicolor*, but there is one exception: two haplotypes which in 2004 had disappeared from their 2001 location, were now recorded more than 400 m apart from it (Fig. 4e).

## Discussion

Here we examined, whether the coexistence of the two ecologically similar [1]*Cataglyphis *species *C. bicolor *and *C. mauritanica *could be due to differences in their life histories. The coexistence of ecologically equivalent or at least similar species of ants is not as uncommon as one might expect it to be in the light of the competitive exclusion principle [3]. Nevertheless, the large amount of studies dealing with the coexistence of such species have focused on niche differentiations within the foraging realm (temporal avoidance: [32-36]; specialization on differently distributed food items: Davidson, [37-39]; differences in worker size: [37,38,40-46]; microhabitat preferences: [47,48]). As there are no differences between *C. bicolor *and *C. mauritanica *in all these respects [1], we asked whether differences in the life histories of the two species might account for the observed coexistence. This is the more likely as the two species differ in the numbers of their queens per colony, with *C. bicolor *and *C. mauritanica *being monogynous and polygynous, respectively. The difference might go hand in hand with different dispersal strategies. Wide-ranging dispersal due to long-distance mating flights is typical for monogynous ants [19,49], whereas in polygynous ants new nests are often founded by budding [19,20,50-52]. In turn, this difference in queen dispersal strategies might lead to different ways of colonizing and occupying temporarily unstable environments. The areas populated by *C. bicolor *and *C. mauritanica *are repeatedly used for growing wheat, where the plowing together with the possible use of insecticides could lead to dramatic breakdowns of *Cataglyphis *populations. In this case the observed heterospecific populations could be a short-term phenomenon during the growing phase of the populations of *C. bicolor *and *C. mauritanica*, when competition for food and/or nest sites is still low. By studying dispersal strategies of both species of ants and recording habitat longevity we tested the hypothesis that the coexistence of the two ecologically similar species of *Cataglyphis *ants reflects a transitional phase occurring within a constantly changing environment.

### Dispersal strategies

Due to the high genetic population viscosity of *C. mauritanica *(Fig. 1 and 4a), the dispersal distances of the queens of this species must be rather short. Most likely such short dispersal distances are caused by dependent colony foundation via budding. As in *C. bicolor *the variability of the mtDNA genes is rather low, the results obtained along the 250-km transect are not as telling in this species as they have been in *C. mauritanica*. In *C. bicolor *most of the colonies shared the same haplotype (Haplotype 1 in Fig. 2). However, a closer look at the distribution of rare haplotypes as they occurred in most of the focus areas provides a clearer view: These rare *C. bicolor *haplotypes were restricted to 2–4 nests located close to each other (see Table 3 and Fig. 4e). If colony foundation occurred by budding, one would have expected that these haplotypes formed large clusters, as it was observed in *C. mauritanica*. We therefore conclude that *C. bicolor *queens found their colonies independently. Later these colonies might become polydomous, but they do not spread over as large areas as they do in *C. mauritanica*.

In both *C. bicolor *and *C. mauritanica *the distribution of haplotypes did not change within a 3-year period. It was only in *C. bicolor *that two of the 7 haplotypes occurred more than 400 m away from their former positions (haplotypes 4 and 7, Fig. 4e). Unfortunately, we cannot say whether the colonies exhibiting these haplotypes have been new foundations from beyond our test area, or whether they have moved by 400 m during the 3-year period.

Far-ranging mating flights in *C. bicolor *males have already been observed from colonies located 60 km south of our Kasserine test area (area of the Oued Kebir, R. Wehner and S. Wehner, unpublished data). In the present account we could observe for the first time, budding in *C. mauritanica*. About 20 workers left a nest and dug a new nest about 30 m apart from the former one. As the excavation of the new nest occurred, 4 dealated queens were waiting at the old nest entrance. After about 4 hours of digging, one of the queens was carried by a worker to the new nest, while the remaining 3 queens stayed in the old nest. Hence, while *C. bicolor *performs mating flights, *C. mauritanica *at least can disperse by budding. These behavioural observations are directly supported by our genetic data. As expected for long migration distances due to mating flights with independent colony foundation, the population of *C. bicolor *is genetically unstructured (Fig. 2), and *C. bicolor *nests belonging to the same haplotype do not form large clusters (Fig. 4e). As expected for short migration distances due to dependent nest founding by budding, the population of *C. mauritanica *is genetically structured (Fig. 1), and *C. mauritanica *nests belonging to the same haplotype form large clusters (Fig. 4a). In conclusion, whereas *C. bicolor *and *C. mauritanica *do not exhibit differences in their foraging characteristics, they markedly differ in their life histories, in colony structure, and the dispersal strategies of their queens.

### Habitat longevity

Do these differences explain the coexistence of the two species? In independent colony founding interspecific and intraspecific competition can cause habitat saturation due to high dispersal risk and low nest founding success rates. Models developed by Nonacs [53] and Pamilo [54] assume high dispersal risks as a key factor leading to polygyny. This assumption was supported by Seppä et al. [55], who reported that in boreal ants, which exhibit facultative polygyny, habitat age is correlated with nest site limitations and the number of queens per nest. In at least 4 out of 5 ant species the number of queens per nest increased with the age of the habitat. Usually, due to the low risk of dependent colony founding ants dispersing by budding such as the pest species *Linepithema humilis *and *Solenopsis invicta *are known to out-compete other species efficiently [19]. All these arguments should let one assume that the monogynous *C. bicolor *is driven to extinction whenever it has to compete with the polygynous *C. mauritanica*. Nevertheless, coexisting populations of both species are not rare, but wherever we found both species inhabiting the same areas, these areas were used for agriculture. Human interference has been shown to increase the densities of populations of two coexisting species of paper wasps, as manmade structures provide the wasps with additional nesting sites [56]. However, in the present account the agriculture could destroy pre-existing *Cataglyphis *nests and by that could cause habitat instability. We therefore propose the hypothesis that the coexistence of *C. bicolor *and *C. mauritanica *does not reflect a stable situation but that it is rather a transitional state during an ongoing re-colonization process occurring within unstable habitats.

In order to test this hypothesis, i.e. to check whether human plowing disequilibrates the habitats in which both *Cataglyphis *species occur sympatrically, we mapped the nests of either species within the very same areas in the years 2001 and 2004. During this 3-year period, 40.2 per cent of the 37.8-ha area under observation had been used agriculturally. The number of nests of both species decreased slightly in those areas that had not been used for agriculture in-between. This slight decrease might be due to either habitat saturation or to the exceptionally hot summer of 2003. However, on those areas that had been used for growing wheat the number of nests decreased dramatically (Fig. 3).

The instability of the habitat occupied by *C. bicolor *and *C. mauritanica *as well as the different life histories, especially the different dispersal strategies, of the two species make the following scenario most likely:

After having been used for farming, the highland steppe regions of central Tunisia are almost free of *Cataglyphis *ants. Afterwards the areas formerly used for human agricultural activity are re-colonized by *C. bicolor *and/or *C. mauritanica*. Due to the restricted sizes of the areas used for agriculture, our data cannot tell, whether *C. bicolor *is the faster a colonizer, the larger the empty habitats are. However, whenever the area is large, its center might first be colonized by *C. bicolor *queens because of their longer dispersal distances. The colonization of the outer parts of the area (or the total area, when it is small) depends on the species composition of the surrounding populations. During the one observed budding process in *C. mauritanica *the new nest was founded 30 m away from the mother nest. Hence, whenever *C. mauritanica *colonies are close by, they should be able to expand into the area by budding. As long as the total nest density is low, single *C. bicolor *queens should also be able to colonize these outer areas. As time proceeds, and if the habitat is not yet used agriculturally again, competition should increase while the populations are growing. In those places in which both species occur sympatrically, the budding polygynous colonies of *C. mauritanica *should then out-compete the monogynous *C. bicolor*. This could happen by competition for food, so that established colonies were displaced, or by competition for nest places, so that new colonies could no longer be established. Due to the long life cycle of ant queens, the latter scenario would lead to an extension of the time frame within which both species could coexist, but nevertheless would finally result in pure *C. mauritanica *populations. However, even if we assume that *C. mauritanica *is the better local competitor, and *C. bicolor *is the faster disperser, a competition-dispersal trade-off alone should be unable to stabilize the coexistence of the two species. It rather seems to be the disturbance via farming that stabilizes the observed sympatry of *C. bicolor *and *C. mauritanica*: As the areas are repeatedly used for growing wheat every 5–8 years (information provided by the local farmers), i.e. before the colonization process has reached a crucial competition stage, the majority of the colonies becomes extinct during the next farming period, and the colonization process can start again. Hence, our final conclusion is that the sympatry of the two *Cataglyphis *species is just a transitional phase during an ongoing colonization process.

## Methods

### Transect

Ants were collected along a 250-km transect, which started close to the Tunisian capital Tunis in the north-eastern part of Tunisia (36.50N 10.13E) and ended south of Kasserine in the south-western part of the country close to the Algerian border (35.13N 8.43E, Fig. 1 and 2). Along the 250-km transect we stopped at every promising area along the road and searched for *Cataglyphis *nests for at least 20 min. Collected ants were stored in absolute ethanol for DNA analysis. We collected *C. mauritanica *workers from 28 nests and *C. bicolor *workers from 35 nests. In addition 109 ants of different *C. mauritanica *nests and 84 ants of different *C. bicolor *nests were collected along small-scale transects at 6 focus areas (each with a size 1.6 – 12.3 × 10^5 ^m^2^). The focus areas were located along the route from Kasserine to Feriana over a distance of about 10 km (Fig. 3a). In order to map the nest positions systematically we scoured the areas along strait parallel lines with a distance of 4 m between the lines. Geographic coordinates were recorded by GPS at all nests from which ants were collected. By using these GPS coordinates we were able to revisit the very same places three years later for remapping the nest distribution and for checking any agricultural use that had been made of the area during the previous three years.

### DNA analysis

From all samples that had been collected in the years 2001 and 2004 we extracted DNA probes of the alitrunks of single ants by using the CTAB method [57] with minor modifications. Proteinase K (20 mg/ml) was used instead of mercaptoethanol. For the analysis of mtDNA the 3'end within the Cytochrome Oxidase 1 gene (CO1) was amplified using the primer COI-RLR (5'-ttgattttttggtcatccagaagt-3'[58]). This sequence corresponds to position 2492 in the complete honeybee mitochondrial genome [59]. For the 5'end within the Cytochrome Oxidase 2 gene (CO2) we used the primer Croz-COII (5'-ccacaaatttctgaacattgacc-3'), which together with COI-RLR amplifies a sequence of about 1520 bp including the leucine tRNA and an intergenic spacer. To enhance the sequence reaction in the inner part of the region we designed an internal primer pair

COIF2 (5'-gcyagattcattcattgatttcctc-3', position 2929)

and COIIR1 (*C. mauritanica*: 5'-taggagaatttgarttttgtagag-3')

or COIIR1bic (*C. bicolor*: 5'-tgggagaatttgaattttgaagtg-3') amplifying 500 of the internal base pairs.

PCR amplifications were carried out in 50 μl reaction volumes containing 1× Buffer A, 0.5 μl DMSO, 0.2 mM each dNTP, 10 pM each primer, about 50 ng DNA, and 1 unit Taq (Promega) with a PTC 100 (MJ Research) for 40 cycles (94°C, 75s, 43°C, 75s, 72°C, 135s) after an initial 180s denaturation step at 95°C and with final extension at 72°C for 300s. PCR reactions were purified with the QIAquick PCR Purification Kit (Qiagen) under conditions specified by the manufacturer. PCR products were sequenced using the ABI-PRISM Dye Terminator Cycle Sequencing Ready Reaction Kit (ABI-Perkin Elmer) in 10 μl reaction volumes following the manufacturer's instructions and run on an ABI 3100 DNA sequencer.

Chromatograms were first checked by eye for base call accuracy and then aligned individually with the opposite strand from the same individual using the program Sequencher™, and sequences were examined for sequence agreement. Finally all sequences were checked for internal stop codons to exclude possible pseudogenes from analysis. All sequences were submitted to GenBank (see Table 1).

### Phylogeography

Sequences alignment and creation of haplotype networks were performed with ClustalX [60] which uses the Neighbour Joining method of Saitou and Nei [61].

Due to the extensive length polymorphism of the intergenic spacer (*C. bicolor*: 76–106 bp, *C. mauritanica*: 23–35 bp) we could not find any satisfying alignment. Furthermore the position of the primer COIIR1 very close to the leucine tRNA gene led to less precise sequencing of this gene. Hence for calculating the networks the non-coding region and the neighbouring leucine tRNA were excluded.

Phylogeographic analysis of the large-scale transect was conducted as described in [27] by calculating the geographic center of every clade (GCC, Fig. 1) of the haplotype tree by averaging the latitude and longitude over all individuals that belong to this clade. Then by averaging latitude and longitude of all samples we calculated the center of the whole studied population (GCP, Fig. 1) and compared for each individual the distances from the GCC and from the GCP. Within a genetically structured population individuals should reveal shorter distances to the center of their clades than to the center of the whole population.

As an additional test for spatial differentiation (association between genetic and geographic distances) geographical distances were taken as the minimum linear distance between sampling sites and the significance of correlation between genetic and geographical distances was assigned by a Mantel test (10000 permutations;[30]).

In all cases in which we had sufficient numbers of nests of different haplotypes per focus area, we checked for clumped or random distributions of identical haplotypes. Distances between nests of identical haplotypes and between nests of different haplotypes were analyzed by the Mantel (10000 permutations [30]). In order to account for effects of polydomy, we excluded nests that were less than 20 m apart from their neighbours from the analysis.

Haplotype networks computed for the sequence data of either the CO1 region or the CO2 region did not lead to any contradicting node. There was no difference in variability between the CO1-coding and the CO2-coding regions with a maximum sequence divergence of 1.1% (CO1) versus 2.2% (CO2) within 25 *C. bicolor *ants and 5.8% (CO1) versus 4.9% (CO2) within 28 *C. mauritanica *ants (partition homogeneity test: p = 0.75). Therefore we were able to run the analyses for both regions together.

## Competing interests

The author(s) declare that they have no competing interests.

## Authors' contributions

MK collected the ants and carried out the molecular genetic studies. RW participated in the design of the study. Both authors together drafted the manuscript, read, and approved the final manuscript.
